# Supplementary material for: Genome-wide identification and characterisation of HOT regions in the human genome
Source: BMC Genomics. 2016 Sep 15;17:733. doi: 10.1186/s12864-016-3077-4 (PMC5025555; doi:10.1186/s12864-016-3077-4)
Supplement: Supplementary file 1 — Supplementary materials. (PDF 2308 kb) [file 12864_2016_3077_MOESM1_ESM.pdf]

Supplementary materials for

Genome-wide identification and characterisation of HOT regions in the human genome

Hao Li[1], Feng Liu[1], Chao Ren[1], Xiaochen Bo[1*], Wenjie Shu[1*]

[1]Department of Biotechnology, Beijing Institute of Radiation Medicine, Beijing 100850, China

[*]Corresponding author. To whom correspondence should be addressed. Tel & Fax: +86 10 68210077 66932211; Email: shuwj@bmi.ac.cn. Correspondence may also be addressed to: boxc@bmi.ac.cn.

Supplementary materials

Results

HOT regions in ESCs

Further characterisation of the ESC HOT regions revealed that they contain many features of LOT regions but at a considerably larger scale. Previous reports have demonstrated that chromatin modifiers are enriched in enhancer regions. In the present study, we found that the levels of enhancer markers, including histone modifications H3K27ac and H3K4me1 [1, 2] and DNase I hypersensitivity [3], in HOT regions significantly exceed the levels in LOT regions. Similar results were observed for active markers, such as H3K9ac. Interestingly, the permissive histone marker H2AZ was significantly depleted in HOT regions, whereas the repressive marker H4K20me1 was significantly enriched in HOT regions. Strikingly, compared to LOT regions, HOT regions were simultaneously enriched with both permissive histone marker H3K4me3 and repressive marker H3K27me3 signals; these signals are thought to play an important role in pluripotency by silencing developmental genes in ESCs while keeping them poised for activation upon differentiation [4, 5].

RNA polymerase II can transcribe enhancers and produce noncoding RNAs that contribute to enhancer activity [6-10]. We measured the levels of RNA polymerase II in HOT and LOT regions to determine the effects of these regions on transcriptional control. RNA polymerase II was highly enriched in HOT regions relative to LOT

regions, which was consistent with RNA signalling levels (Fig. S3A). This result helps to explain why HOT regions drive high-level expression of their associated genes compared to LOT regions (Fig. S3B). Our results suggest that HOT regions could be involved in regulating RNA polymerase II activities and could therefore affect gene expression. Thus, HOT regions may harbour features resembling those of recently identified enhancer RNAs that can contribute to enhancer function [6-8, 11-15].

To further investigate the factors that constitute HOT and LOT regions, we compiled chromatin immunoprecipitation-sequencing (ChIP-seq) data for 13 different chromatin regulators and 30 TFs in ESCs from the ENCODE project [3] (Figs. S3C and S3F). Notably, a broad spectrum of chromatin regulators (12 out of 13, 92%) and transcription regulators (26 out of 30, 87%) that are responsible for cell growth, tissue development, cell cycle progression and developmental events, including ATF2, POU5F1, HDAC2, HDAC6, and PHF8, are especially enriched in ESC HOT regions relative to LOT regions. In contrast, four chromatin regulators and TFs (CTCF, RAD21, BCL11A, and MAFK) were significantly enriched in ESC LOT regions relative to HOT regions. Recent studies have revealed that CTCF and RAD21 co-occupy many genomic targets of pluripotency factors in ESCs to play key roles in the control of pluripotency and cellular differentiation [16, 17]. Strikingly, SUZ12 and JARID1 were differentially depleted within HOT and LOT regions. SUZ12, a subunit of PRC2, maintains pluripotency in ESCs by repressing developmental genes that are preferentially activated during ESC differentiation [18]. Recent studies from multiple model

organisms, including corn fungus, yeast, *C. elegans*, *Drosophila*, zebrafish, and mice, have demonstrated that JARID1 proteins, as histone H3K4 demethylases, play key roles in development and differentiation [19-21].

Distinct sequence signatures of HOT regions

To gain insight into characteristic sequence features of HOT regions, we studied the enrichment of known TF motifs in HOT and LOT regions using HOMER [22]. Both the genome and the LOT/HOT regions were used as backgrounds in the motif scanning within HOT/LOT regions, respectively. Overall, 226 out of 542 (41.7%) TFs with known motifs exhibited significantly enriched binding in HOT or LOT regions. Of these 226 TFs, 59 (26.1%) TFs exhibited specifically enriched binding within HOT regions relative to the expectations based on the backgrounds of both genome and LOT regions. The majority of these TFs play important roles in development, including MYB, MZF1, TCF7, ZBTB7A/B, HNF4A, POU1F1, PAX2, SRF, XBP1, EGR3 and CREB1, as well as in cell proliferation and differentiation, including RORA, E4F1, MECOM, SP1, RREB1 and FOXM1. Thirty-four (15.2%) factors exhibited significantly enriched binding in LOT regions relative to expectations based on the backgrounds of both the genome and HOT regions. Strikingly, 12 of these 34 TFs (*p*-value = 0.0012, binomial test) were housekeeping TFs associated with the regulation of transcription (NFE2L1, REST, TCF4, NFYC, YY1), protein binding (NFKB1, RBPJ, SMAD4), TF activity (RELA), negative regulation of granulocyte differentiation (RUNX1), multicellular organismal development (TCF12), and the nucleus (SP3).

Additionally, we found that a small fraction (8 out of 226, 3.5%) of TFs exhibited specifically enriched binding in both HOT and LOT regions relative to the expectations based on the two backgrounds. These TFs play important roles in development and differentiation, including POU3F2, TCF3, SPY, and MYC, as well as housekeeping roles such as response to oxidative stress, including FOXO1 and NFE2L2.

Identifying HOT regions in many cell types

To characterise the HOT regions in as many human cells as possible, we applied a uniform processing pipeline to create a catalogue of HOT regions based on DNase-seq data from 349 samples, including 154 cell and tissue types studied under the ENCODE Project [3, 23]. We identified an average of 8,036 HOT regions per cell type (range 2,405 to 19,753, Table S2), spanning on average ~1.7% of the genome. In total, we identified 59,986 distinct HOT regions along the genome, collectively spanning 18.8%. To assess the rate of discovery of new HOT regions, we performed a saturation analysis as described in a previous study [3] and predicted saturation at approximately 107,184 (standard deviation = 8,608) HOT regions and 774,925,252 bp (standard deviation = 33,534,434) (40.9%) of genome coverage (Fig. S2A). This result indicates that we have discovered more than half of the estimated total HOT regions.

Of these 59,986 HOT regions, 287 (0.5%) localise to UTRs defined by GENCODE, and a collective 9% lie within promoter (n = 4,039, 6.7%) and exon (n = 1,391, 2.3%) regions. Among the remaining HOT regions, 56.8% (n = 34,090) and 33.6% (n = 20,179)

are positioned in intronic and intergenic regions, respectively (Fig. S2B).

Gene Ontology (GO) analysis of HOT regions

We next performed GO analysis on HOT region-associated genes (HOT genes). This analysis revealed that HOT genes are linked to developmental processes of the respective cell and tissue types (Fig. S3E). To gain further understanding of the transcriptional regulatory circuitry of development, it would be valuable to identify key developmental TFs that control this process. As the majority of HOT genes are involved in developmental processes, we deduced that candidate key developmental TFs could be identified in human cells by identifying HOT genes that encode TFs. We then performed this analysis in all of the 154 human cell types. For cells in which key developmental TFs have already been identified, this analysis captured the vast majority of these factors (Table S3). A catalogue of candidate key developmental TFs for other cell types can be found in Table S4. These candidates will be helpful in deducing the transcriptional regulatory circuitry of diverse human cell types and in further understanding cell development and cell differentiation.

Materials and Methods

Characterisation of HOT Regions

The genome-wide ChIP-seq densities of TF and histone modifications around HOT regions and LOT regions (Figs. S3A, S3C and S3F) were created by mapping reads to

these regions and their corresponding ±5 kb flanking regions. Each HOT/LOT region and its flanking regions were split into 50 equally sized bins. This procedure split all HOT/LOT regions, regardless of their size, into 150 bins. All HOT/LOT regions were then aligned, and the average ChIP-seq density in each bin was calculated to create a genome-wide average in units of reads per kilobase per million (rpkm).

To find sequence motifs enriched in HOT and LOT regions, we analysed the genomic sequences under the DHSs within these regions. HOMER [22] was used with the default parameters to examine whether any of the 542 TFs from TRANSFAC [24], JASPAR [25], and UniPROBE [26] were overrepresented. Overrepresentation was statistically evaluated using three independent background sets: the entire chromosome 20, all the RefSeq transcription start sites (TSSs) (±2.0 kb), and all of the CpG islands annotated in the hg19 genome. A motif was retained only when it was significantly overrepresented ($P \leqslant 0.01$) compared to all of these backgrounds.

Gene Ontology Analysis

For gene ontology (GO) analysis, a subset of 19 data sets, which represented the diversity of cells in the collection used for this study, was first selected. Each HOT region was assigned to the closest genes annotated in GENCODE (V15) by determining the distance from the centre of the HOT region to the TSS of each GENCODE gene. For each cell, the genes associated with HOT regions in that cell and no more than six other cells in the subset were analysed using Database for Annotation, Visualization

and Integrated Discovery (DAVID) [27]. For each cell, the four top scoring categories (i.e., the categories with the lowest *p*-values) were selected for display. A threshold *p*-value score of 10^{-6} was incorporated as a minimum requirement filter for scoring as a top category.

Supplementary figures

Figure S1. Validation of HOT regions in GM12878, HeLas3, HepG2 and K562 cell lines, related to Figure 1.

(A) Distribution of TFBS complexity signal across the 80,326 TFBS-clustered regions in H1 cells. TFBS-clustered regions are plotted in increasing order based on their TFBS complexity signal. HOT regions are defined as the population of TFBS-clustered regions above the inflection point of the curve. (B) Error bar showing the GSC results of HOT/LOT regions versus classical HOT/LOT regions in GM12878, HeLaS3, HepG2, and K562. Red lines indicate the mean and normalised SD of 10,000 bootstrap samples; blue bar indicates the real statistics. (C) ROC curves of the validation of predicted HOT regions, area under roc curve (AUC area) were shown in brackets. (D) The proportion of HOT regions and classical HOT regions containing different numbers of TF ChIP-seq peaks in GM12878, HeLaS3, HepG2, and K562. (E) The distributions of TFBS complexity of HOT regions containing different numbers of TF ChIP-seq peaks in GM12878, HeLaS3, HepG2, and K562. (F) The proportion of motifless binding peaks occurred in experimental HOT regions and predicted HOT regions. (G)

The proportion of motifless HOT regions occurred in experimental HOT regions and predicted HOT regions.

Figure S2. General features of HOT regions in many cell types, related to Figure 1

(A) Saturation analysis of HOT regions. We modelled saturation for element count and length using a Weibull distribution ($r^2 \geqslant 0.995$) and predicted saturation at approximately 107,184 (sd = 8,608) and 774,925,252 (sd = 33,534,434) for count and length, respectively. The cell line estimation of 95% saturation is 222 and 154 for count and length, respectively. (B) Distribution of 59,986 HOT regions and 301,322 LOT regions with respect to GENCODE gene annotations. Promoter regions are defined as the first region located within 1 kb upstream and downstream of a GENCODE TSS.

Figure S3. Identification and characterisation of HOT regions in ESCs, related to Figure 1

(A) Metagene representations of the mean ChIP-seq signal for the indicated DNaseI, RNA polymerase II (RNAPII), histone modifications and RNA-seq across LOT (blue) and HOT (red) regions. Metagenes are centered on the TFBS-clustered region (5863 bp and 11,890 bp for LOT and HOT regions, respectively) with 5 kb surrounding each TFBS-clustered region. (B) Gene expression level of HOT-specific genes (red) and LOT-specific genes (blue). (C) Metagene representations of the mean ChIP-seq signal

for the indicated transcription factors, transcriptional cofactors, and chromatin regulators across LOT (blue) and HOT (red) regions. (D) Motif enrichment in HOT and LOT regions comparing with different backgrounds. Heat map showing the most differentially distributed motifs (multiple testing corrected P-value < 0.01) between HOT regions compared with the genome average values (first column), HOT and LOT regions (second column), LOT regions compared with the genome average values (third column), LOT and HOT regions (fourth column). (E) GO terms for HOT-region-associated genes that were closest to its associated HOT regions 19 human cell and tissue types with corresponding p-values obtained by DAVID. (F) Metagene representations of the mean ChIP-seq signal for the additional indicated transcription factors, transcriptional cofactors, and chromatin regulators across LOT (blue) and HOT (red) regions.

Figure S4. Association of HOT regions with functional regulatory elements, related to Figure 2

(A) Examples of known cell-selective experimentally validated distal, non-promoter *cis*-regulatory elements. Shown above each set of DNaseI data are schematics displaying HOT regions relative to the genes they control. (B-D) GSC results between HOT regions and LMRs, UMRs and DMVs, Red lines indicate the mean and normalised SD of 10,000 bootstrap samples; blue bar indicates the real statistics.

Figure S5. GO analysis of super-enhancer and HOT regions, Related to Figure 4

(A) GO analysis of super-enhancer-associated genes and HOT region-associated genes in H1 hESC, CD20, and pancreas cells. The top 10 scoring categories were selected for display. A threshold *p*-value score of 10^{-4} was incorporated as a minimum requirement filter for scoring as a top category.

Supplementary tables

Table S1. H1-hESC TFBS cluster information, related to Figure 1

Table showing the TF complexity cutoffs for HOT regions in H1hESCs: the total number of H1-hESC TFBS clusters is 80,326, the number of HOT regions is 8,533, and the median lengths of HOT and LOT regions are 11,890 bp and 5,863 bp, respectively.

Table S2. Comparison of motifless binding peaks and HOT regions, related to Figure 1

Table showing the detailed information of the comparison of motifless binding peaks and HOT regions in 5 cell types.

Table S3. Information on HOT regions in 154 files, related to Figure 1

Table showing the TF complexity cutoffs for HOT regions, HOT region number, total

number of TFBS clusters and genome coverage in 154 cell lines.

Table S4. Key TF genes, Related to Figure1

Function role and references about Key developmental transcription factor genes.

Table S5. Key developmental transcription factor genes, Related to Figure1

Development associated transcription factor genes identified in 154 cell lines.

Table S6. Repetitive elements in HOT regions, related to Figure 2

Overlap of repeat-masked elements by repeat family for families with more than 2,000 elements overlapping DHSs. Column 1 shows the repeat family; column 2 shows the repeat class. Column 3 shows the average size of elements in the family; column 4 shows the total number of occurrences of elements of the family in the genome. Column 5 indicates the number of repeat families that overlap a HOT region by at least 95%.

Table S7. 1046 validated elements in HOT regions, related to Figure 2

Enrichment of validated elements in HOT and LOT regions. The number of non-VISTA enhancer-associated elements is 373, while the total number of validated elements is 1,046.

Table S8. Genes associated with “gained” HOT regions, related to Figure 5

Table showing 1011, 692, 839, 1547 and 854 expressed genes (RPKM > 1) associated with “gained” HOT regions of ME, NPC, TBL, MSC and IMR90 cells, respectively. GO analysis of these genes in respective cell were also showed.

Table S9. Genes associated with enriched TF genes, related to Figure 5

Table showing 106, 153, 178, 75 and 35 enriched TF genes with RPKM > 1 for ME, NPC, TBL, MSC and IMR90 cells, respectively. GO analysis of these genes in respective cell were also showed.

Table S10. Enrichment of bivalent genes in HOT regions, related to Figure 5

We got totally 3,191 bivalent genes from Zhao et al., 2007 and Pan et al., 2007, and the enrichment was calculated by hypergeometric test.

Table S11. GO analysis of activated HOT regions in H1-derived cells, related to Figure 6

Table showing GO results of genes associated “activated” HOT regions in H1-derived cells.

References

1. Creyghton MP, Cheng AW, Welstead GG, Kooistra T, Carey BW, Steine EJ, Hanna J, Lodato MA, Frampton GM, Sharp PA *et al*: **Histone H3K27ac separates active from poised enhancers and predicts developmental state**. *Proceedings of the National Academy of Sciences of the United States of America* 2010, **107**(50):21931-21936.
2. Rada-Iglesias A, Bajpai R, Swigut T, Brugmann SA, Flynn RA, Wysocka J: **A unique chromatin signature uncovers early developmental enhancers in humans**. *Nature* 2011, **470**(7333):279-283.
3. Consortium TEP: **An integrated encyclopedia of DNA elements in the human genome**. *Nature* 2012, **489**(7414):57-74.
4. Bernstein BE, Mikkelsen TS, Xie X, Kamal M, Huebert DJ, Cuff J, Fry B, Meissner A, Wernig M, Plath K *et al*: **A bivalent chromatin structure marks key developmental genes in embryonic stem cells**. *Cell* 2006, **125**(2):315-326.
5. Azuara V, Perry P, Sauer S, Spivakov M, Jorgensen HF, John RM, Gouti M, Casanova M, Warnes G, Merkenschlager M *et al*: **Chromatin signatures of pluripotent cell lines**. *Nature cell biology* 2006, **8**(5):532-538.
6. Kim TK, Hemberg M, Gray JM, Costa AM, Bear DM, Wu J, Harmin DA, Laptewicz M, Barbara-Haley K, Kuersten S *et al*: **Widespread transcription at neuronal activity-regulated enhancers**. *Nature* 2010, **465**(7295):182-187.
7. Lam MT, Cho H, Lesch HP, Gosselin D, Heinz S, Tanaka-Oishi Y, Benner C, Kaikkonen MU, Kim AS, Kosaka M *et al*: **Rev-Erbs repress macrophage gene expression by inhibiting enhancer-directed transcription**. *Nature* 2013, **498**(7455):511-515.
8. Li W, Notani D, Ma Q, Tanasa B, Nunez E, Chen AY, Merkurjev D, Zhang J, Ohgi K, Song X *et al*: **Functional roles of enhancer RNAs for oestrogen-dependent transcriptional activation**. *Nature* 2013, **498**(7455):516-520.
9. Natoli G, Andrau JC: **Noncoding transcription at enhancers: general principles and functional models**. *Annual review of genetics* 2012, **46**:1-19.
10. Sigova AA, Mullen AC, Molinie B, Gupta S, Orlando DA, Guenther MG, Almada AE, Lin C, Sharp PA, Giallourakis CC *et al*: **Divergent transcription of long noncoding RNA/mRNA gene pairs in embryonic stem cells**. *Proceedings of the National Academy of Sciences of the United States of America* 2013, **110**(8):2876-2881.
11. Lai F, Orom UA, Cesaroni M, Beringer M, Taatjes DJ, Blobel GA, Shiekhattar R: **Activating RNAs associate with Mediator to enhance chromatin architecture and transcription**. *Nature* 2013, **494**(7438):497-501.
12. Orom UA, Derrien T, Beringer M, Gumireddy K, Gardini A, Bussotti G, Lai F, Zytnicki M, Notredame C, Huang Q *et al*: **Long noncoding RNAs with enhancer-like function in human cells**. *Cell* 2010, **143**(1):46-58.
13. Ling J, Ainol L, Zhang L, Yu X, Pi W, Tuan D: **HS2 enhancer function is blocked by a transcriptional terminator inserted between the enhancer and the promoter**. *The Journal of biological chemistry* 2004, **279**(49):51704-51713.
14. Kaikkonen MU, Spann NJ, Heinz S, Romanoski CE, Allison KA, Stender JD, Chun HB, Tough DF, Prinjha RK, Benner C *et al*: **Remodeling of the enhancer landscape during macrophage activation is coupled to enhancer transcription**. *Molecular cell* 2013, **51**(3):310-325.

15. Mousavi K, Zare H, Dell'orso S, Grontved L, Gutierrez-Cruz G, Derfoul A, Hager GL, Sartorelli V: **eRNAs promote transcription by establishing chromatin accessibility at defined genomic loci**. *Molecular cell* 2013, **51**(5):606-617.
16. Nitzsche A, Paszkowski-Rogacz M, Matarese F, Janssen-Megens EM, Hubner NC, Schulz H, de Vries I, Ding L, Huebner N, Mann M *et al*: **RAD21 cooperates with pluripotency transcription factors in the maintenance of embryonic stem cell identity**. *PloS one* 2011, **6**(5):e19470.
17. Kagey MH, Newman JJ, Bilodeau S, Zhan Y, Orlando DA, van Berkum NL, Ebmeier CC, Goossens J, Rahl PB, Levine SS *et al*: **Mediator and cohesin connect gene expression and chromatin architecture**. *Nature* 2010, **467**(7314):430-435.
18. Lee TI, Jenner RG, Boyer LA, Guenther MG, Levine SS, Kumar RM, Chevalier B, Johnstone SE, Cole MF, Isono K *et al*: **Control of developmental regulators by Polycomb in human embryonic stem cells**. *Cell* 2006, **125**(2):301-313.
19. Shi Y: **Histone lysine demethylases: emerging roles in development, physiology and disease**. *Nature reviews Genetics* 2007, **8**(11):829-833.
20. Nottke A, Colaiacovo MP, Shi Y: **Developmental roles of the histone lysine demethylases**. *Development (Cambridge, England)* 2009, **136**(6):879-889.
21. Benevolenskaya EV: **Histone H3K4 demethylases are essential in development and differentiation**. *Biochemistry and cell biology* 2007, **85**(4):435-443.
22. Heinz S, Benner C, Spann N, Bertolino E, Lin YC, Laslo P, Cheng JX, Murre C, Singh H, Glass CK: **Simple combinations of lineage-determining transcription factors prime cis-regulatory elements required for macrophage and B cell identities**. *Molecular cell* 2010, **38**(4):576-589.
23. Qu H, Fang X: **A brief review on the Human Encyclopedia of DNA Elements (ENCODE) project**. *Genomics, proteomics & bioinformatics* 2013, **11**(3):135-141.
24. Matys V, Kel-Margoulis OV, Fricke E, Liebich I, Land S, Barre-Dirrie A, Reuter I, Chekmenev D, Krull M, Hornischer K *et al*: **TRANSFAC and its module TRANSCompel: transcriptional gene regulation in eukaryotes**. *Nucleic acids research* 2006, **34**(Database issue):D108-110.
25. Portales-Casamar E, Thongjuea S, Kwon AT, Arenillas D, Zhao X, Valen E, Yusuf D, Lenhard B, Wasserman WW, Sandelin A: **JASPAR 2010: the greatly expanded open-access database of transcription factor binding profiles**. *Nucleic acids research* 2010, **38**(Database issue):D105-110.
26. Robasky K, Bulyk ML: **UniPROBE, update 2011: expanded content and search tools in the online database of protein-binding microarray data on protein-DNA interactions**. *Nucleic acids research* 2011, **39**(Database issue):D124-128.
27. Huang da W, Sherman BT, Lempicki RA: **Systematic and integrative analysis of large gene lists using DAVID bioinformatics resources**. *Nature protocols* 2009, **4**(1):44-57.

Figure S1

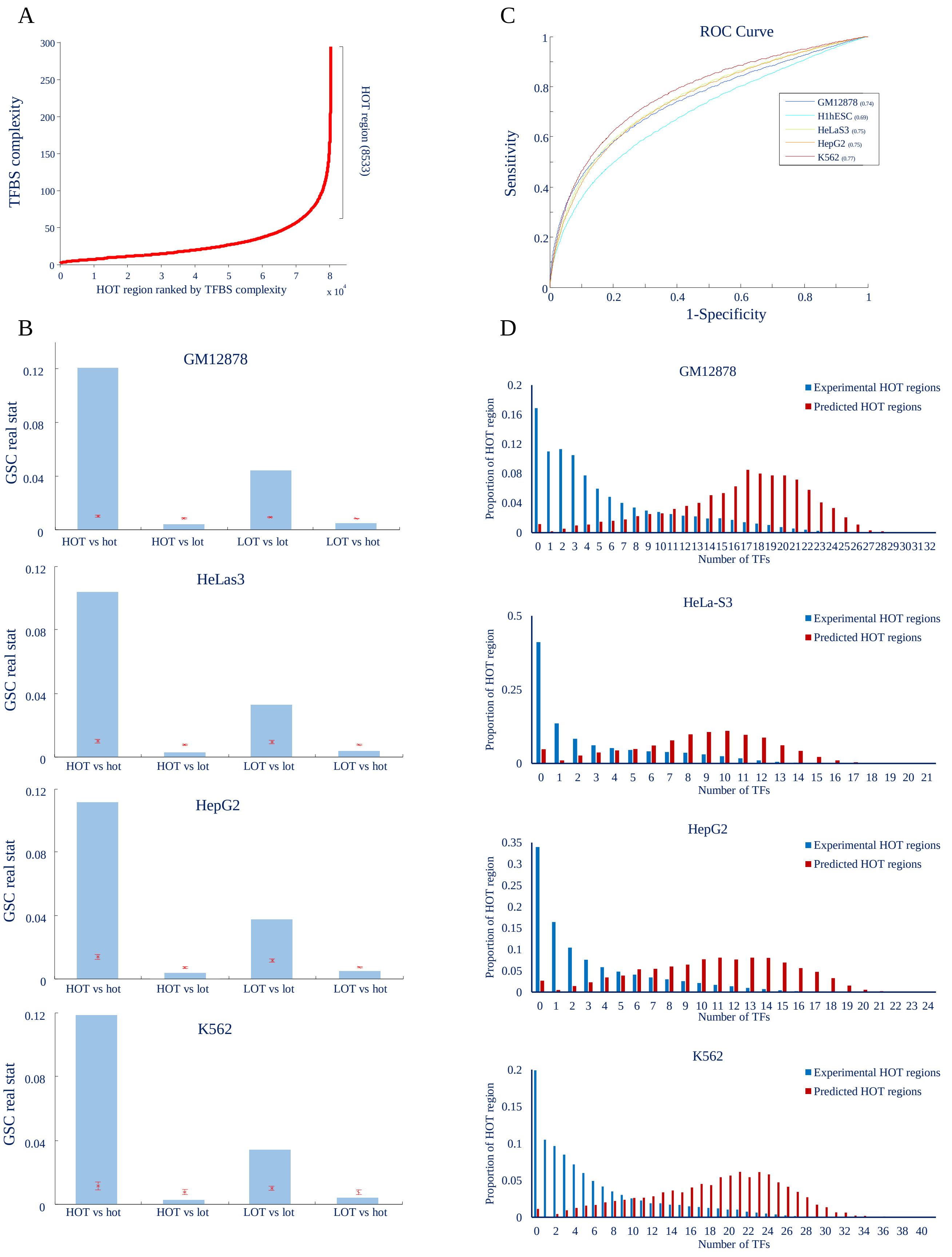
A
TFBS complexity
HOT region ranked by TFBS complexity
x 10^4
HOT region (8533)
C
ROC Curve
Sensitivity
1-Specificity
GM12878 (0.74)
H1hESC (0.69)
HeLaS3 (0.75)
HepG2 (0.75)
K562 (0.77)
B
GM12878
HeLas3
HepG2
K562
GSC real stat
HOT vs hot
HOT vs lot
LOT vs lot
LOT vs hot
D
GM12878
HeLa-S3
HepG2
K562
Experimental HOT regions
Predicted HOT regions
Proportion of HOT region
Number of TFs

E

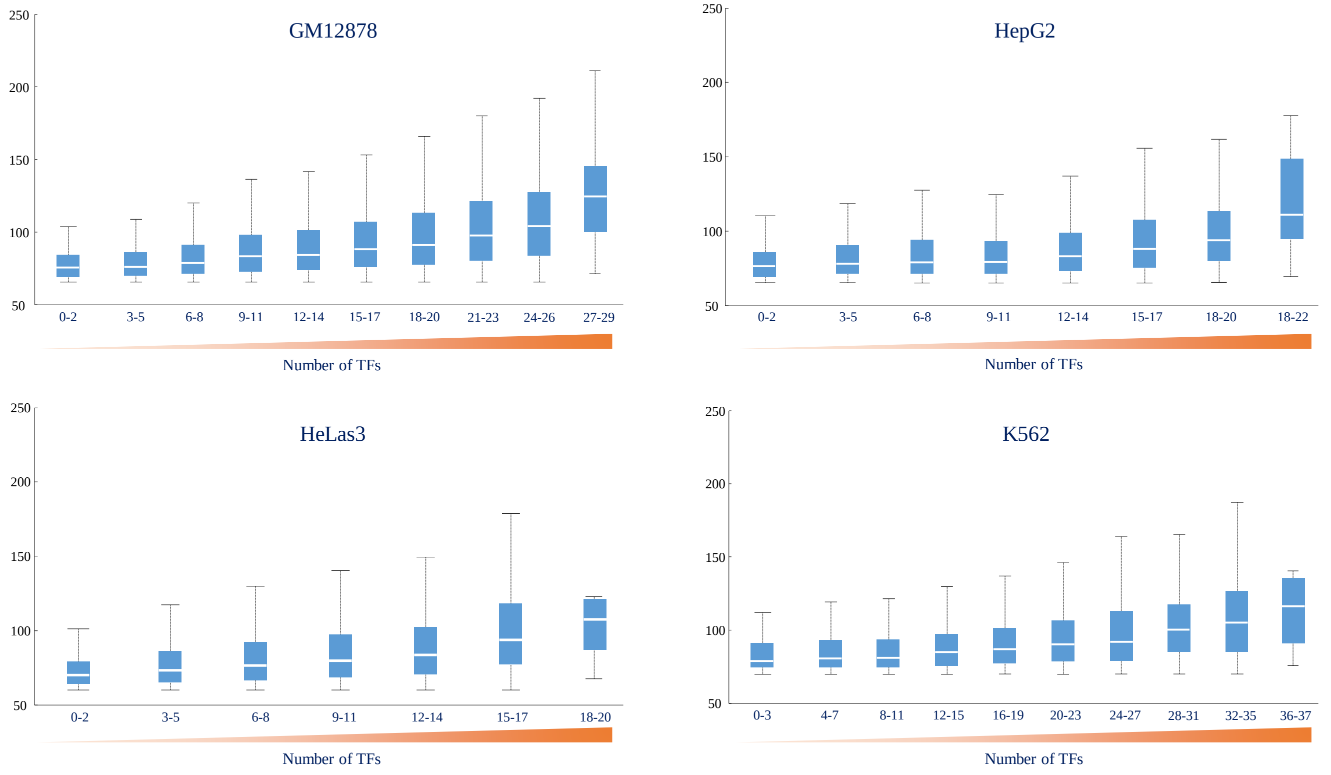
GM12878
250
200
150
100
50
0-2
3-5
6-8
9-11
12-14
15-17
18-20
21-23
24-26
27-29
Number of TFs
HepG2
250
200
150
100
50
0-2
3-5
6-8
9-11
12-14
15-17
18-20
18-22
Number of TFs
HeLas3
250
200
150
100
50
0-2
3-5
6-8
9-11
12-14
15-17
18-20
Number of TFs
K562
250
200
150
100
50
0-3
4-7
8-11
12-15
16-19
20-23
24-27
28-31
32-35
36-37
Number of TFs

F

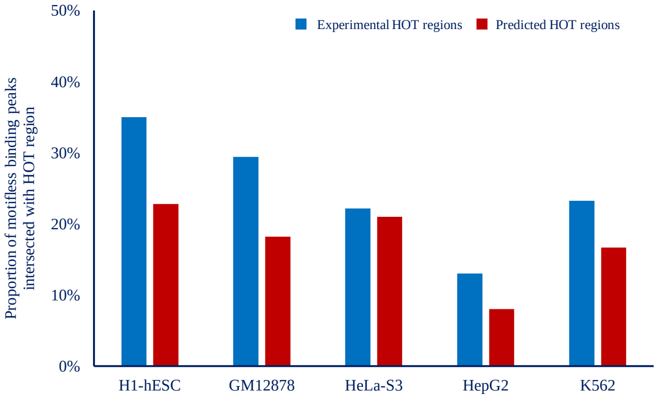
Proportion of motifless binding peaks intersected with HOT region
50%
40%
30%
20%
10%
0%
Experimental HOT regions
Predicted HOT regions
H1-hESC
GM12878
HeLa-S3
HepG2
K562

G

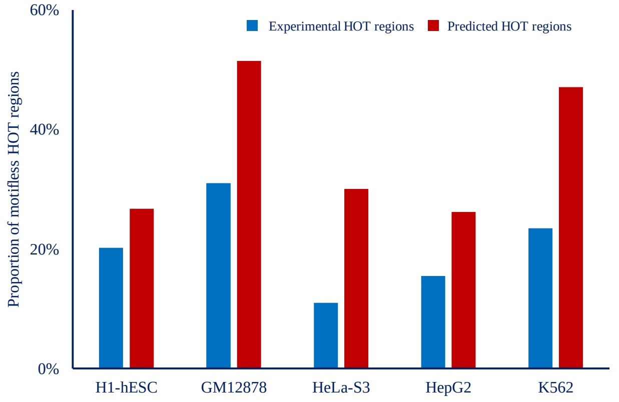
Proportion of motifless HOT regions
60%
40%
20%
0%
Experimental HOT regions
Predicted HOT regions
H1-hESC
GM12878
HeLa-S3
HepG2
K562

Figure S2

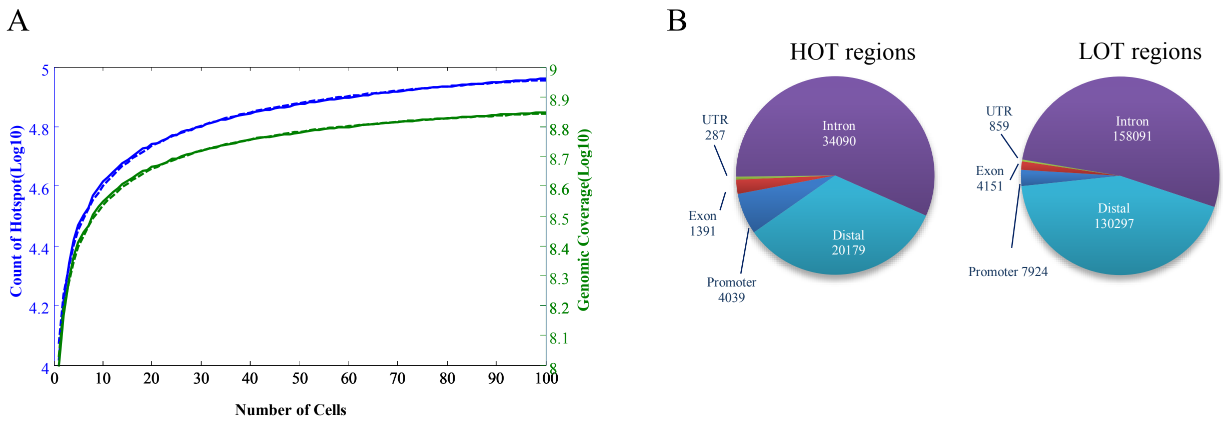

A
B
Count of Hotspot(Log10)
Genomic Coverage(Log10)
Number of Cells
HOT regions
LOT regions
Intron
34090
Distal
20179
UTR
287
Exon
1391
Promoter
4039
Intron
158091
Distal
130297
UTR
859
Exon
4151
Promoter 7924

Figure S3

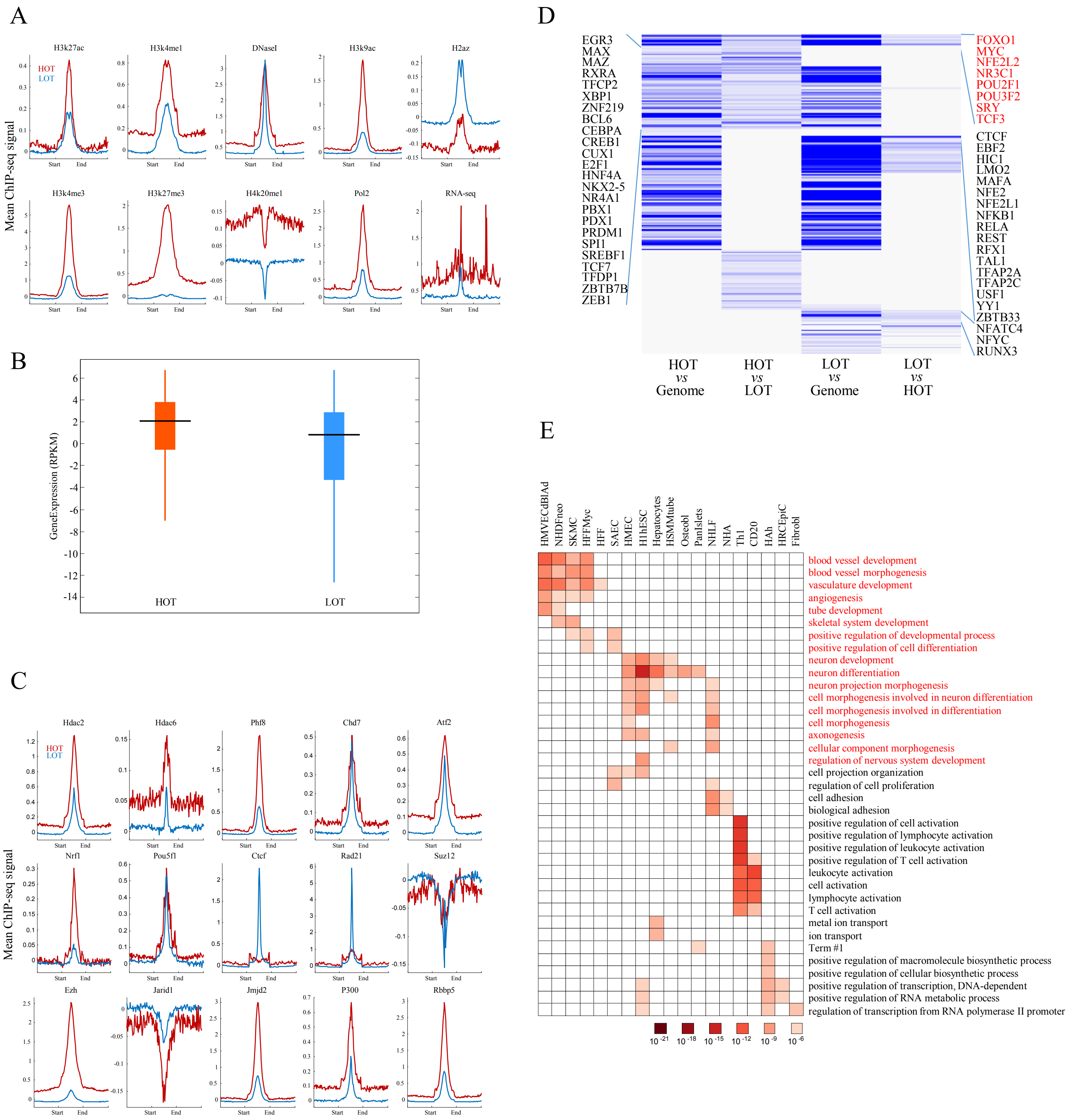
A
H3k27ac
H3k4me1
DNaseI
H3k9ac
H2az
HOT
LOT
Mean ChIP-seq signal
H3k4me3
H3k27me3
H4k20me1
Pol2
RNA-seq
Start
End
B
GeneExpression (RPKM)
HOT
LOT
C
Hdac2
Hdac6
Phf8
Chd7
Atf2
Nrf1
Pou5f1
Ctcf
Rad21
Suz12
Ezh
Jarid1
Jmjd2
P300
Rbbp5
Mean ChIP-seq signal
D
EGR3
MAX
MAZ
RXRA
TFCP2
XBP1
ZNF219
BCL6
CEBPA
CREB1
CUX1
E2F1
HNF4A
NKX2-5
NR4A1
PBX1
PDX1
PRDM1
SPI1
SREBF1
TCF7
TFDP1
ZBTB7B
ZEB1
FOXO1
MYC
NFE2L2
NR3C1
POU2F1
POU3F2
SRY
TCF3
CTCF
EBF2
HIC1
LMO2
MAFA
NFE2
NFE2L1
NFKB1
RELA
REST
RFX1
TAL1
TFAP2A
TFAP2C
USF1
YY1
ZBTB33
NFATC4
NFYC
RUNX3
HOT vs Genome
HOT vs LOT
LOT vs Genome
LOT vs HOT
E
HMVECdBlAd
NHDFneo
SKMC
HFFMyc
HFF
SAEC
HMEC
H1hESC
Hepatocytes
HSMMtube
Osteobl
PanIslets
NHLF
NHA
Th1
CD20
HAh
HRCEpiC
Fibrobl
blood vessel development
blood vessel morphogenesis
vasculature development
angiogenesis
tube development
skeletal system development
positive regulation of developmental process
positive regulation of cell differentiation
neuron development
neuron differentiation
neuron projection morphogenesis
cell morphogenesis involved in neuron differentiation
cell morphogenesis involved in differentiation
cell morphogenesis
axonogenesis
cellular component morphogenesis
regulation of nervous system development
cell projection organization
regulation of cell proliferation
cell adhesion
biological adhesion
positive regulation of cell activation
positive regulation of lymphocyte activation
positive regulation of leukocyte activation
positive regulation of T cell activation
leukocyte activation
cell activation
lymphocyte activation
T cell activation
metal ion transport
ion transport
Term #1
positive regulation of macromolecule biosynthetic process
positive regulation of cellular biosynthetic process
positive regulation of transcription, DNA-dependent
positive regulation of RNA metabolic process
regulation of transcription from RNA polymerase II promoter
10 -21
10 -18
10 -15
10 -12
10 -9
10 -6

Figure S3

F

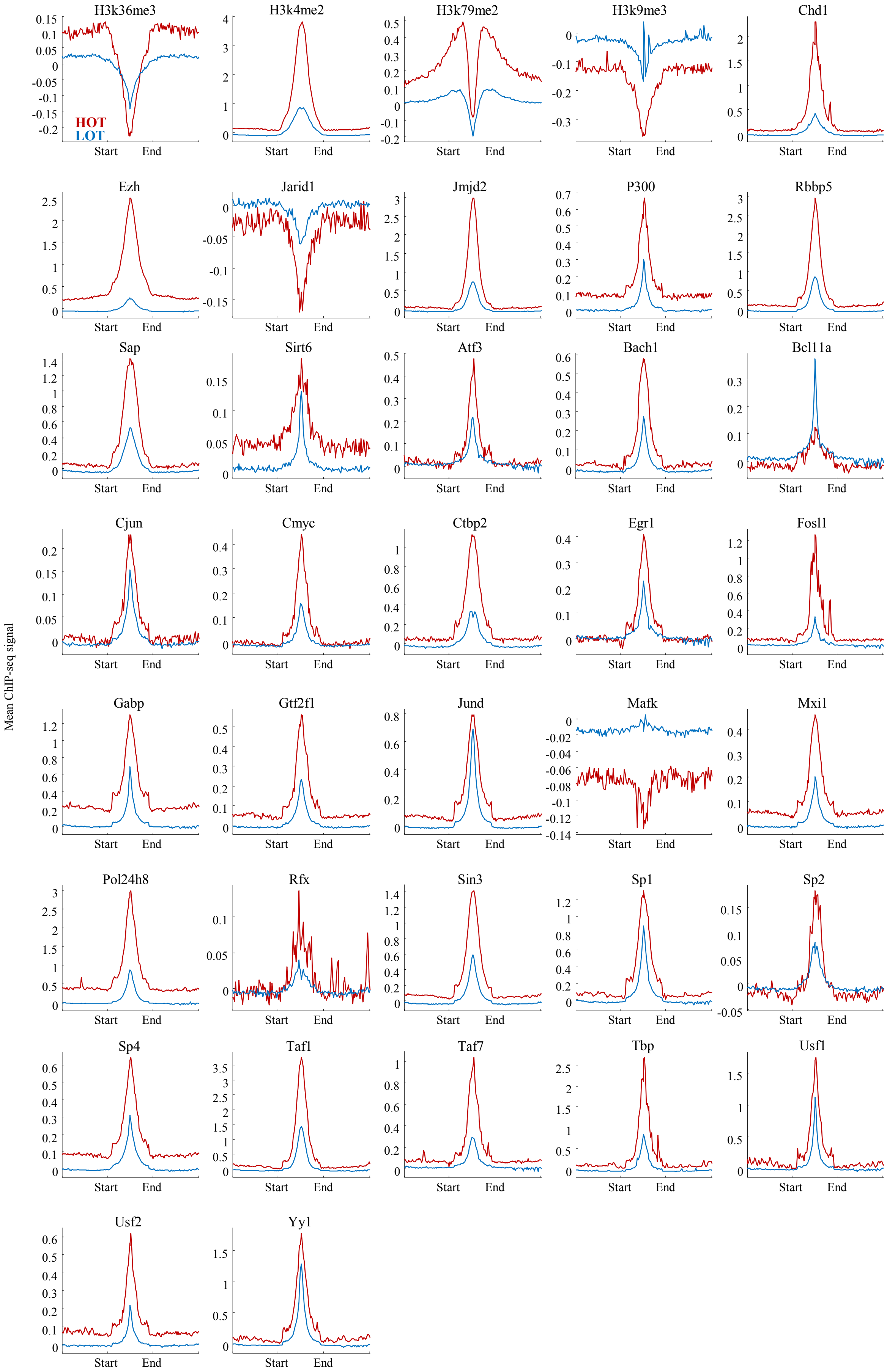
H3k36me3
H3k4me2
H3k79me2
H3k9me3
Chd1
HOT
LOT
Ezh
Jarid1
Jmjd2
P300
Rbbp5
Sap
Sirt6
Atf3
Bach1
Bcl11a
Cjun
Cmyc
Ctbp2
Egr1
Fosl1
Gabp
Gtf2f1
Jund
Mafk
Mxi1
Pol24h8
Rfx
Sin3
Sp1
Sp2
Sp4
Taf1
Taf7
Tbp
Usf1
Usf2
Yy1
Start
End
Mean ChIP-seq signal

Figure S4

A

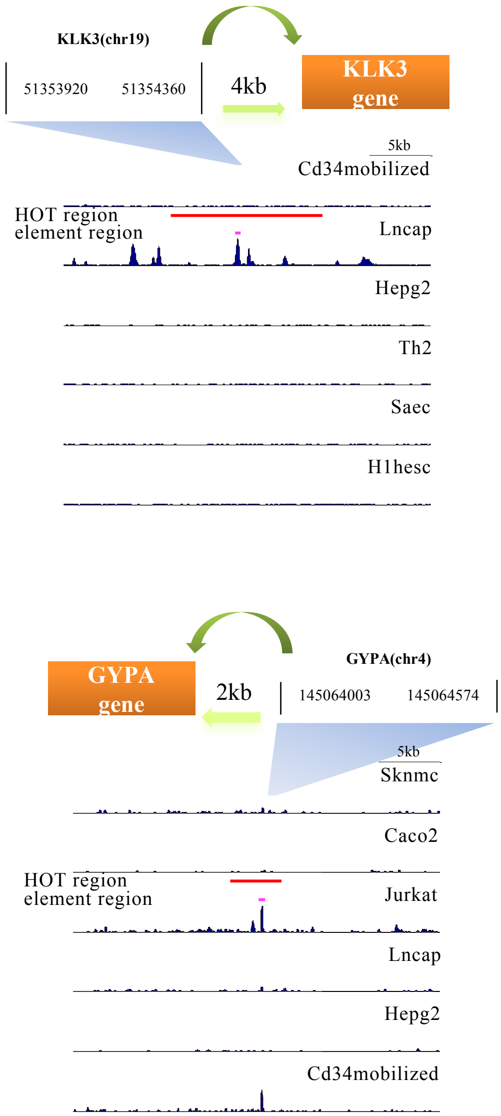

B

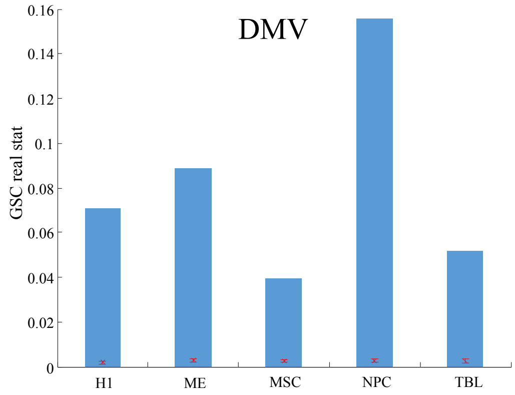

C

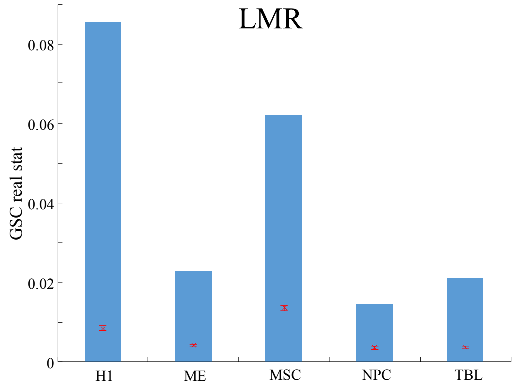

D

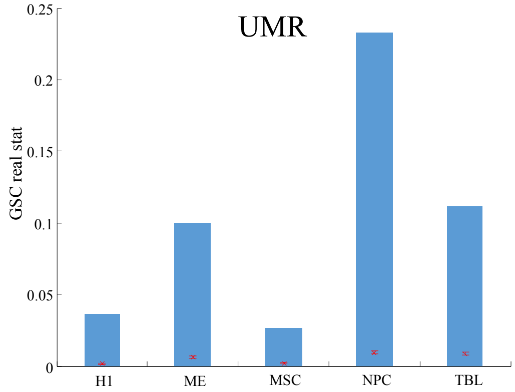

Figure S5

A

SuperEnhancer		HOT region	
**H1**			
**GO term**	**P value**	**GO term**	**P value**
axon guidance	8.98E-06	neuron differentiation	1.25E-15
axonogenesis	3.17E-05	neuron development	2.42E-10
neuron projection development	5.40E-05	cell morphogenesis involved in differentiation	4.38E-10
cell morphogenesis involved in neuron differentiation	7.57E-05	regulation of nervous system development	1.31E-09
neuron projection morphogenesis	9.27E-05	cell morphogenesis involved in neuron differentiation	1.76E-09
negative regulation of transcription	2.79E-04	regulation of neurogenesis	3.47E-09
regulation of neuron differentiation	3.22E-04	embryonic organ morphogenesis	5.01E-09
negative regulation of RNA metabolic process	3.42E-04	sensory organ development	1.00E-08
cell morphogenesis involved in differentiation	3.84E-04	neuron projection morphogenesis	1.03E-08
cell projection morphogenesis	3.97E-04	axonogenesis	1.30E-08
**CD20**			
**GO term**	**P value**	**GO term**	**P value**
immune response	3.46E-09	leukocyte activation	1.71E-13
antigen processing	5.05E-07	cell activation	3.31E-12
cell activation	2.12E-06	lymphocyte activation	3.50E-12
regulation of lymphocyte activation	6.12E-06	T cell activation	4.33E-08
positive regulation of cell activation	7.23E-06	positive regulation of T cell activation	2.14E-07
regulation of cell activation	9.59E-06	positive regulation of lymphocyte differentiation	2.26E-07
positive regulation of lymphocyte activation	1.10E-05	regulation of lymphocyte differentiation	6.09E-07
regulation of programmed cell death	1.37E-05	lymphocyte differentiation	7.21E-07
regulation of cell death	1.49E-05	immune response	7.24E-07
regulation of leukocyte activation	2.28E-05	positive regulation of T cell differentiation	8.59E-07
**Pancreas**			
**GO term**	**P value**	**GO term**	**P value**
regulation of transcription	3.32E-06	neuron differentiation	2.91E-08
endocrine pancreas development	1.12E-05	positive regulation of transcription	9.40E-07
pancreas development	1.12E-05	neuron development	3.93E-06
regulation of cell proliferation	3.76E-05	ion transport	1.04E-05
positive regulation of cell proliferation	5.39E-05	cation transport	1.33E-05
positive regulation of nitrogen compound metabolic process	3.93E-04	regulation of transcription	2.20E-05
positive regulation of macromolecule biosynthetic process	4.62E-04	positive regulation of transcription	2.38E-05
positive regulation of cellular biosynthetic process	7.46E-04	positive regulation of RNA metabolic process	3.03E-05
positive regulation of biosynthetic process	8.64E-04	regulation of cell development	5.74E-05
positive regulation of nucleobase	8.94E-04	cell motion	7.63E-05
